# ^57^Fe Mössbauer Spectroscopy as a Tool for Study of Spin States and Magnetic Interactions in Inorganic Chemistry

**DOI:** 10.3390/molecules26041062

**Published:** 2021-02-18

**Authors:** Ernő Kuzmann, Zoltán Homonnay, Zoltán Klencsár, Roland Szalay

**Affiliations:** 1 Laboratory of Nuclear Chemistry, Institute of Chemistry, Eötvös Loránd University, 1117 Budapest, Hungary; homonnay@ludens.elte.hu (Z.H.); klencsar.zoltan@ek-cer.hu (Z.K.); szalayr@chem.elte.hu (R.S.); 2 Center for Energy Research, 1121 Budapest, Hungary

**Keywords:** molecular magnetism, Mössbauer spectroscopy, spin crossover, iron(II/III) phthalocyanines, magnetic exchange interactions

## Abstract

In this mini-review of our research group’s activity, the application of ^57^Fe Mössbauer spectroscopy in studies of electronic structure, coordination environment, and magnetic interactions in an interesting series of Fe(II/III) compounds selected is discussed. We selected two prominent phenomena that arose during investigations of selected groups of compounds carried out at different periods of time: (1) very high magnetic hyperfine fields observed at low temperatures; (2) changes in the oxidation state of the central iron atom of complexes in the solid state during interactions with gaseous O_2_/H_2_O mixtures, resulting in spin crossover (SCO).

## 1. Introduction

One very interesting field of modern molecular chemistry is the preparation and investigation of properties of specially designed single molecules, metal clusters, or groups of molecules that exhibit cooperative effects and extended magnetic interactions [1,2,3]. These characteristics are important for applications such as molecular spintronics, quantum technology, organometallic frameworks, and 2D materials [4,5]. The high-performance information processing devices of the future, such as high-capacity data storage equipment, require compounds that can form molecular-level clusters or thin films that can be deposited on a dielectric base. 

Single-molecule magnets (SMMs) known to date [3,6,7,8,9] usually contain clusters of paramagnetic ions bound to simple ligands (oxide, alkoxide, and halide), surrounded by organic molecular ligands. In the case of SMMs, there are also organometallic compounds [6] that show superparamagnetism below a certain temperature corresponding to the molecular size range. Many SMM compounds contain iron, such as the so-called {Fe^III^_4_}, {Fe^III^_8_}, and {DyFe^III^_3_} compounds.

**Figure 1:**
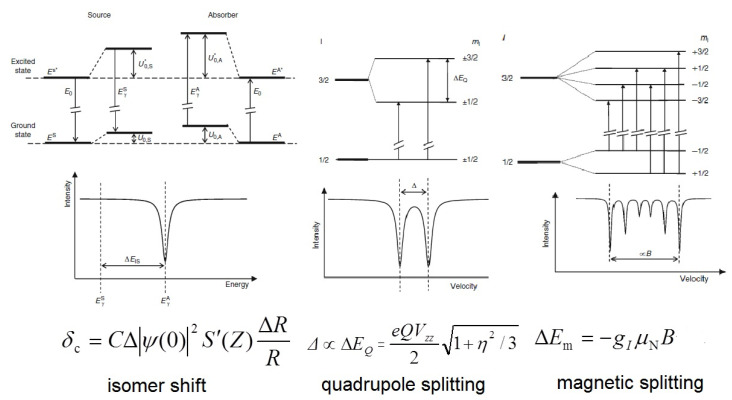
Energy level diagrams for hyperfine interactions at 3/2→1/2 nuclear spin transition and a schematic representation of the corresponding Mössbauer parameters in the spectrum. In the formulas, |Ψ(0)|^2^ is the *s* electronic density, *R* is the radius of the nucleus, *S’(Z)* is the Shirley factor, *Q* is the nuclear quadrupole moment, *Vzz* is the principal component of electric field gradient, *η* is the asymmetry parameter, *g_I_* is the nuclear gyromagnetic factor, μ_N_ is the nuclear magneton and *B* is the magnetic flux density.

Mössbauer spectroscopy is truly preferred as the most informative technique for the study of such compounds [10,11,12,13,14]. In nuclear physics today, Mössbauer spectroscopy [15,16,17,18,19] has become a widely used method for materials analysis, because it can be used to measure hyperfine interactions between electrons and nuclei with an accuracy of 13-15 orders of magnitude. Measurements of Mössbauer spectral parameters provide information on local electron density, local electric field gradients, and the local effective magnetic field (Figure 1). For iron-containing inorganic compounds, solid state phases, materials, or alloys, ^57^Fe Mössbauer spectroscopy has a great advantage over other methods because it allows researchers to obtain information about (1) the metal oxidation state; (2) the spin state; (3) the iron center microenvironment; and (4) the absence/presence and origin of hyperfine magnetic fields. Thus, Mössbauer spectroscopy is suitable for detecting and distinguishing magnetic coupling (ferromagnetic, antiferromagnetic, and ferrimagnetic) between metal centers. This task can be achieved by examining the exchange interactions between coupled centers using an external magnetic field [20]. In addition, spin relaxation processes in Fe-containing compounds can be monitored.

**Figure 2:**
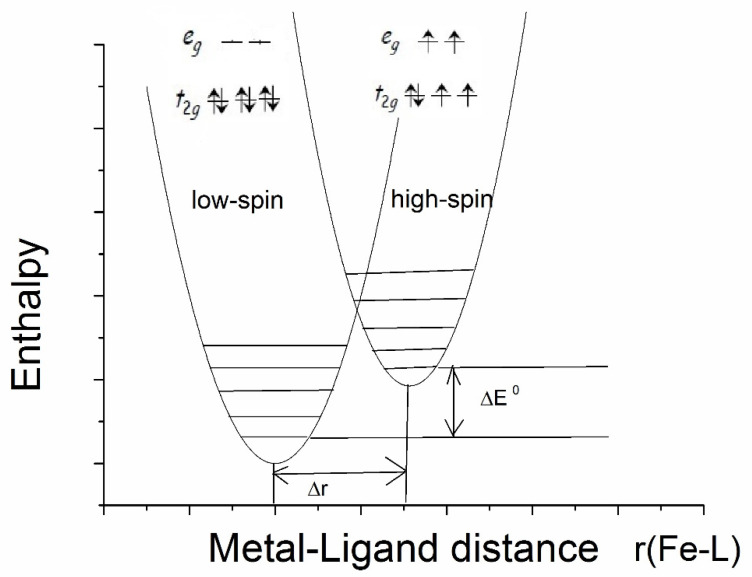
Distribution of electrons on a 3*d* shell in low-spin and high-spin configurations in Fe^II^ and their enthalpy curves as a function of iron-ligand distance.

Spin transition may occur in transition metal complexes with an octahedral inner sphere of the central atom if the metal ion can acquire a low-spin (LS) state, as well as in high-spin (HS) states with a small enthalpy difference. The most commonly studied spin transition molecules are Fe^II^ compounds [21]. Spin transition can be initiated by changing the temperature, pressure, or visible light exposure. The schematic distribution of the electrons on the *3d* shell of Fe^II^ in the low-spin and high-spin configurations and their associated enthalpy curves is shown in Figure 2, with vibrational levels indicated. At low temperatures, the low-enthalpy, low-spin state is more stable, but if the temperature exceeds a certain value, the high-spin electron structure will become the more thermodynamically stable state. Spin transition is a molecular phenomenon that depends on intermolecular interactions. The whole crystal lattice is involved in the process of the spin transition of the central iron ion. Mössbauer spectroscopy is well-positioned as a diagnostic method to determine spin transitions in iron complexes [21,22]. Since both Fe^II^ and Fe^III^ are distinguishable by Mössbauer measurements, different isomer shift and quadrupole splitting values are characteristic for low- and high-spin compounds. Namely, the lower isomer shift is characteristic of the LS states for both Fe^II^ and Fe^III^, while the quadrupole splitting was found to be lower in LS Fe^II^ compounds and higher in LS Fe^III^ compounds as compared to the relevant HS states [21]. For example, the isomer shifts typical of LS Fe^II^ compounds are *δ* = −0.1 to 0.2 mm/s, the quadrupole splitting is Δ = 0.2 to 1.9 mm/s, and while the typical isomer shifts of HS Fe^II^ compounds are *δ* = 0.9 to 1.3 mm/s, the quadrupole splitting is in the range Δ = 2.0–2.7 mm/s at room temperature [16,19]. Notably, the Mössbauer parameter ranges usually do not overlap each other.

In this mini-review, we present some of our research group’s recent results on spin transitions and magnetic coupling in oxygenated iron(II)/(III) phthalocyanines and two-coordinate linear iron(II) complexes, which were studied with extensive use of Mössbauer spectroscopy.

## 2. Extreme High Hyperfine Field and Ferromagnetic Interaction in Linear, Two-Fold Coordinated Single-Molecule Magnetic Iron Complexes

**Chart 1:**
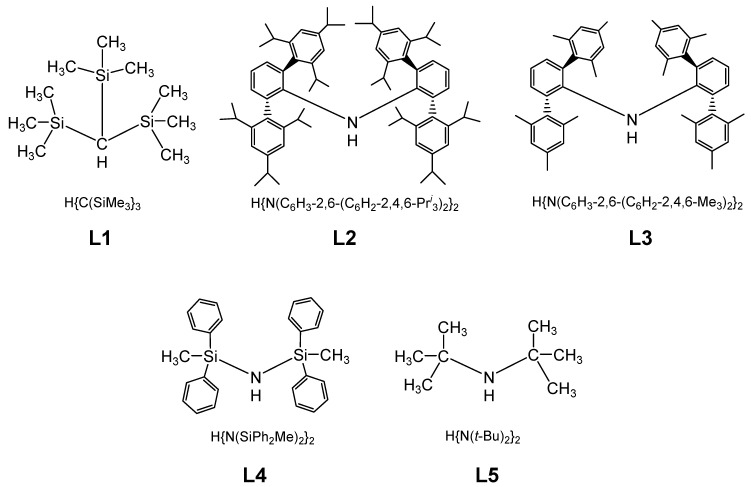
Chemical structures used in the preparation of sterically protected linear and quasi-linear iron(II) amido and methanido complexes.

**Figure 3:**
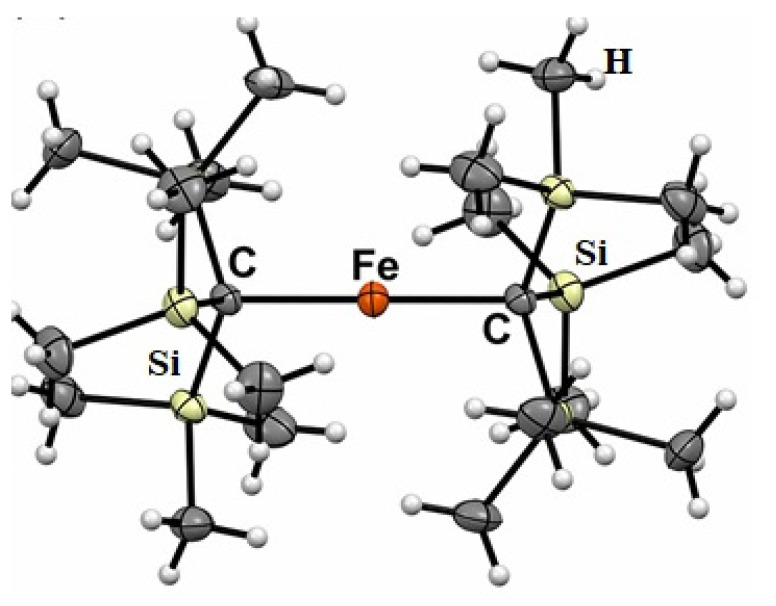
Molecular structure of Fe^II^[C(SiMe_3_)_3_]_2_ (Fe(**L1**)_2_).

Molecular magnetism, which is the focus of current scientific interest with possible technological aspects, is mostly present in molecules with unusual geometry and bonding anisotropy [1,2,3,23], such as two-coordinate iron(II) complexes with linear geometry that possess interesting electrical and magnetic properties [24,25,26,27,28,29,30,31,32,33,34]. In order to create such an unusually low coordination number and stabilize low-valent metal centers, a special set of bulky, protective ligands was designed, as shown in Chart 1. These are sterically demanding methanide (**L1**) and amide (**L2**–**L5**) ligands, which become mono-anions upon deprotonation (Chart 1). Thus, complexes of FeL_2_ type were formed, and only one, Fe^II^[C(SiMe_3_)_3_]_2_ (Fe(**L1**)_2_ later), represents a highly air and moisture sensitive organometallic compound [32], the structure of which is presented in Figure 3. The protective ligand is therefore trimethyl(trisilyl)methanide. Other Fe**L**_2_ complexes (L = **L2**–**L5**) are formally low-coordination, number-unstable Werner-type complexes. In this structure, the iron atom forms a linear C-Fe-C array with a 180 °C-Fe-C angle. Three tri-methylated silicon atoms forming an excellent protective shield are bound to two carbon atoms connected to the metal center (Figure 3).

**Figure 4:**
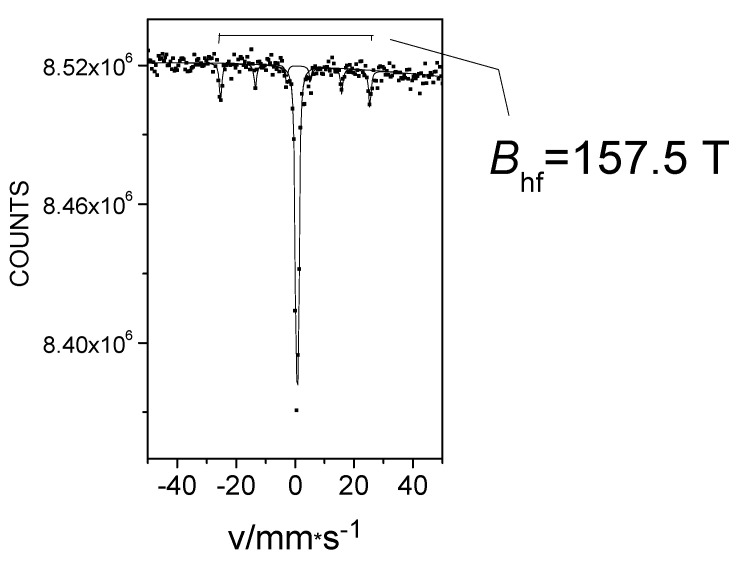
The ^57^Fe Mössbauer spectrum of Fe(**L1**)_2_ at 20 K.

In the Fe^II^[C(SiMe_3_)_3_]_2_ (Fe(**L1**)_2_) molecule, an unusually large ^57^Fe hyperfine magnetic field was detected at low temperatures [26,27,28]. At first, we obtained this Fe(**L1**)_2_ compound and characterized it using ^57^Fe-Mössbauer spectroscopy, which is known for accurate measurement of hyperfine magnetic field strength at the iron site, and by other physical methods that helped to confirm and explain the extremely high magnetic field at the central atom [25,26,33,34]. The results of the geometry optimization and electronic structure determination performed by density functional theory (DFT) calculations were in agreement with the geometry determined by X-ray analysis. Furthermore, the calculations indicated that the HS state of iron was energetically favorable. The DFT analysis showed that there was a significant charge transfer from the iron to the two methanide **L1** C(SiMe_3_)_3_^−^ ligands. According to the calculations, the electron configuration of the Fe atom in the test compound was [Ar]3*d*^5.83^ 4*s*^0.72^, where the 4s electron density was only slightly polarized and the vast majority of unpaired electrons occupied the 3*d* subshell [25,26]. In the ^57^Fe-Mössbauer spectrum of Fe(**L1**)_2_ at 20 K, a component with an extraordinary large internal magnetic field strength of 157.5 T was observed (Figure 4) [25,26].

The high internal magnetic field observed in Fe(**L1**)_2_ was surprising because the contribution of the Fermi contact field to the internal magnetic field generally remains below 55 T in conventional iron compounds [18]. It is known [20] that hyperfine magnetic fields measured at the site of the iron nucleus have three main contributors: the Fermi contact field (*B*_c_), the dipole field, (*B*_d_) and the orbital field (*B*_L_). Compared to the Fermi contact field, the contribution of the dipole field is usually small and often has an opposite direction. As with many transition metals, the orbital moment is “frozen” in the 3*d* shell of the iron in the crystal lattice due to its interaction with the crystal fields of conventional geometries—tetragonal, octahedral, etc. As a result, such “frozen” orbitals do not normally contribute to the hyperfine field. The occasionally occurring small orbital contributions in iron compounds may be related to the spin-orbit coupling in systems with less traditional geometries such as tetrahedral, trigonal, or linear. The extremely high magnetic field value detected by the Mössbauer measurements may be explained by the effect of non-frozen 3*d* orbital contributions in structures with unusual geometry [26]. Based on the alternating current (AC) magnetic susceptibility measurements, the compound Fe(**L1**)_2_ can be viewed as a single-molecule magnet with a *U* = | D | *S*^2^ = 181 cm^−1^ energy barrier [30]. In clusters of SMM molecules containing multiple iron atoms, the exchange interaction within the cluster is essential [6]. it is not clear whether or not an exchange interaction-based magnetism could occur in an Fe(**L1**)_2_ single-molecule magnet with only a single iron atom within a complex. To study this possibility, Mössbauer measurements in an externally applied magnetic field were taken to distinguish between different magnetic couplings. 

**Figure 5:**
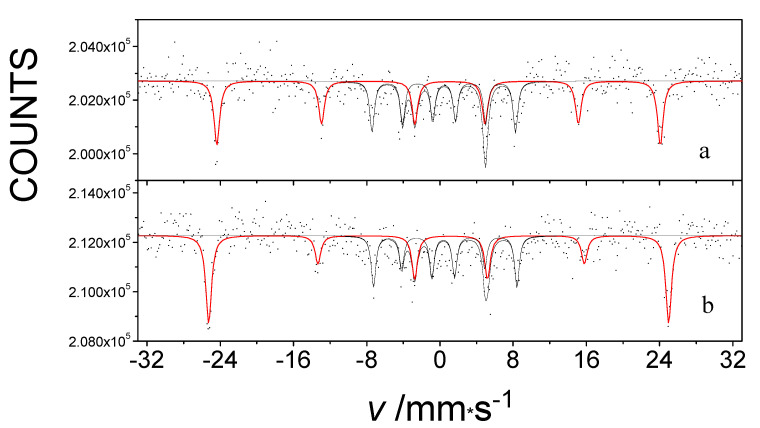
The ^57^Fe Mössbauer spectra of an impure Fe(**L1**)_2_ system measured at 5 K without an external magnetic field (**a**) and in a 5 T *B*_ext_ external magnetic field (**b**) applied parallel to the gamma rays.

Figure 5 shows the ^57^Fe Mössbauer spectra of Fe(L1)_2_ measured at 5 K, without an applied magnetic field and under an external magnetic field of 5T. Both spectra consist two sextets, which provide evidence for the presence of two phases in that particular sample. Sextet S1, which has an unusually high magnetic field, is the fingerprint of the iron atom linearly coordinated by the two carbon atoms in Fe(**L1**)_2_. The inner sextet, S2, belongs to the ferrihydrite (Fe_2_O_3_∙0.5H_2_O) present as a second phase in the sample, which was an impurity that most likely originated as a result of minor hydrolysis of the main compound by moisture. The internal magnetic field manifested by the S1 sextet was found to have increased by the value of the applied external magnetic field with which it interacted. At the same time, the spectrum of the ferrihydrite remained unchanged [35] (Figure 5). Thus, the low-temperature exchange interaction in Fe(**L1**)_2_ is confirmed to be of a ferromagnetic type, in agreement with the hysteresis clearly observed in magnetization measurements of the same system [33].

**Figure 6:**
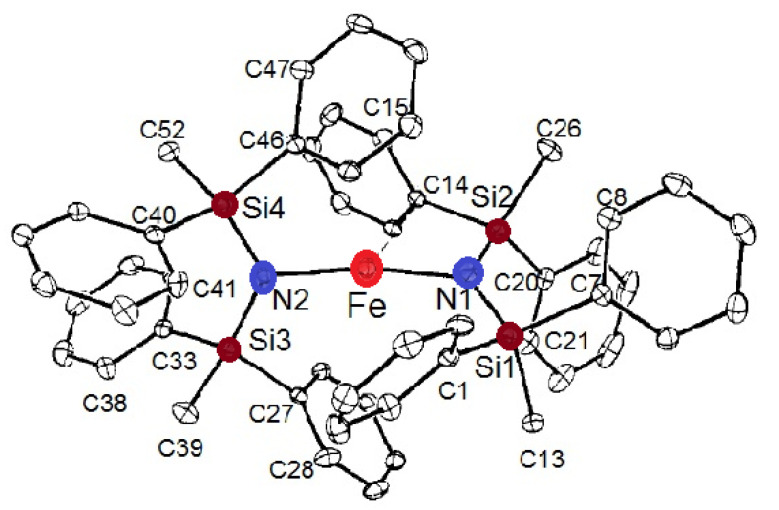
The molecular structure of Fe^II^[N(SiPh_2_Me)_2_]_2_ (Fe(**L4**)_2_).

Further exploration of this topic of unusually high hyperfine internal magnetic fields at low temperatures led to the preparation of other related compounds in which the C-atom in methanide L1 was replaced by a nitrogen atom in a series of similarly sterically hindered amides **L2**–**L5** (Chart 1). New valuable information was obtained from a series of similarly unstable two-coordinate Fe^II^ complexes. The structure of one of them, Fe^II^[N(SiPh_2_Me)_2_]_2_ (Fe(**L4**)_2_), is shown in Figure 6. It is interesting to note that in the complexes Fe(**L2**)_2_ and Fe(**L5**)_2_, both of which have extremely bulky protective metal center ligands (Chart 1), the N-Fe-N angle warrants their linear description, while linearity is lost for the other two amides, Fe(**L3**)_2_ and Fe(**L4**)_2_ (Table 1).

**Figure 7:**
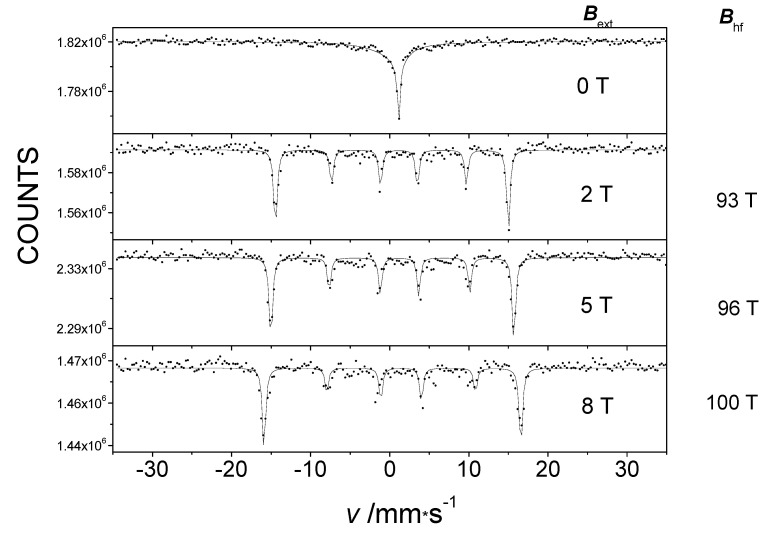
The 5 K Mössbauer spectra of pure Fe(**L4**)_2_ without an external magnetic field and in an external magnetic field *B*_ext_ applied parallel to the direction of the gamma rays, showing the *B*_hf_ hyperfine field.

In the absence of an applied magnetic field, the ^57^Fe Mössbauer spectrum of Fe(**L4**)_2_ appears as a broadened single line (Figure 7, top). However, when an external magnetic field was applied, an unusually high internal magnetic field [34] was detected in the Mössbauer spectra, similar to the case of the compound Fe(**L1**)_2_. The Mössbauer spectra exhibited the classic magnetic 6-line splitting depicted in Figure 7. This observation also provides evidence for the considerable magnitude of the orbital momentum on the metal center in the Fe(**L4**)_2_ molecule. The hyperfine magnetic field value was greater than 90 T, much higher than what could be expected from the contribution of the *B*_c_ Fermi contact field and the *B*_d_ dipole field to the hyperfine field for the high-spin state of Fe^II^ in this complex. Consequently, the occurrence of the unusually high non-frozen orbital moment of the corresponding *B*_L_ orbital contribution caused the observed high hyperfine field.

**Figure 8:**
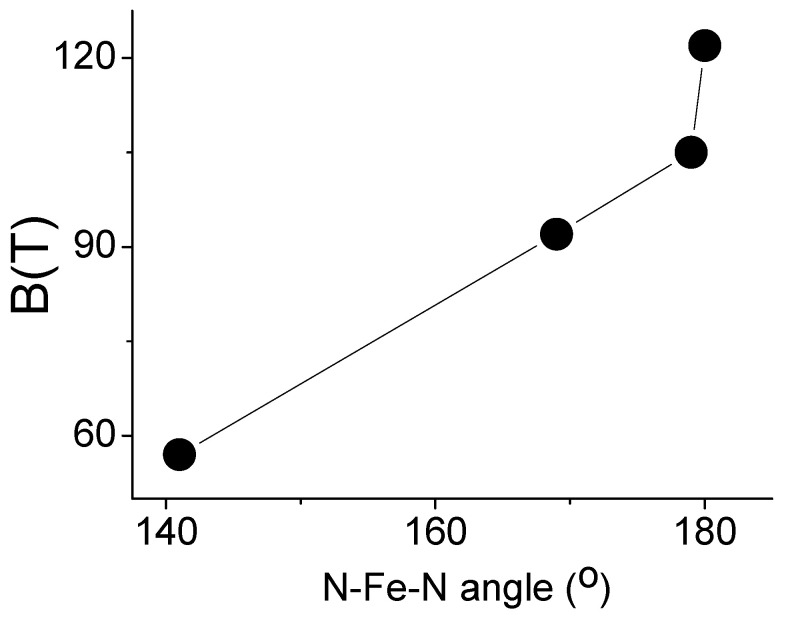
Graph depicting the relationship between the N-Fe-N angle and the magnitude of the internal magnetic field in a series of Fe**L**_2_ amide complexes (Table 1).

Figure 8 shows a correlation between the induced hyperfine magnetic field and the N-Fe-N bond angle in Fe(**L2**)_2_……Fe(**L5**)_2_ molecules [28,30,34]. The value of the hyperfine field increases with an increasing N-Fe-N angle [34]. This can be explained by the fact that the value of the non-frozen orbital moment decreases with the bending of the N-Fe-N structural motif, since the iron atom interacts more with other atoms than it does with nitrogen. Therefore, Fe(**L4**)_2_ is another example of a low-coordination iron complex where quasi-free-ion magnetism occurs through a non-frozen orbital moment.

The effect of the applied magnetic field on the Mössbauer spectra of Fe(**L4**)_2_ indicates a ferromagnetic exchange interaction [34] similar to that shown above for Fe(**L1**)_2_ [33]. The magnetization curves support the notion that magnetism is based on the exchange interaction and correlate well with the presented Mössbauer spectroscopy data [34].

In conclusion, we observed an unusually high internal hyperfine magnetic field on the central atom centers in a series of the linear and quasi-linear crystalline two-coordinated iron (II) methanido and amido complexes. This field can be observed either by simple cooling of the compound to low temperatures (as in Fe(**L1**)_2_) or by applying an external magnetic field with an order of magnitude weaker than that of the generated internal hyperfine field (as in Fe(**L2**-**L5**)_2_ molecules). Several factors were found to affect the observed magnetic coupling: (1) ligand field symmetry/geometry, (2) ligand field strength, (3) composition of the compound (type of ligand used), (4) overall molecular geometry with respect to the linearity of the two-coordinate moiety, and (5) spin–orbit coupling and orbital contribution stemming from the iron center.

Further investigations of similar systems are necessary to better understand the effect of these factors on the very large *B*_hf_ observed in such compounds.

## 3. Temperature-Dependent Spin Transitions and Antiferromagnetic Interaction in Iron Phthalocyanines Loaded with Oxygen in the Solid State

**Figure 9:**
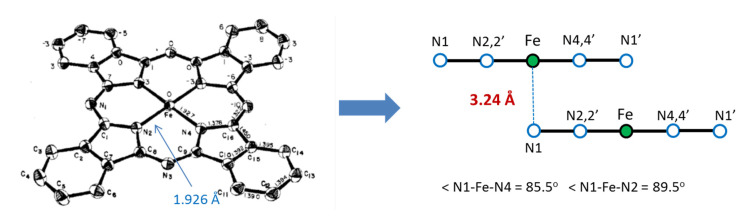
Molecular structure of β-FePc (left panel) and its crystal packing, showing the layered structure and some details of its geometry (from ref. [37]).

As a result of our collaboration with scientists from Drexel University (Philadelphia, PA, USA), we found a new method for the encapsulation of ferromagnetic iron carbides into carbon nanotubes by pyrolysis of the iron(II) phthalocyanines, FePc, in both the α-and β-forms [36]. This complex has a layered structure, with an Fe^II^ center in a square-planar environment with very short Fe-N(ring) bonds of 1.926 Å (Figure 9). The FePc units form a slipped π-stacked arrangement in the crystal where Fe is in very long Fe-N distance (3.24 A) from the N1 atom of an adjacent molecule [37].

As new materials, iron carbides obtained this way have received considerable attention in recent years for potential technological applications in the development of magnetic data storage materials, as toners in xerography, and as a component of ferrofluids [38]. We carried out investigations to better understand the formation mechanism of iron carbides encapsulated in carbon nanoparticles, with the most intriguing results presented below. 

In several experiments, solid iron(II) phthalocyanine was reacted with oxygen at room temperature and slightly above room temperature under dry and wet conditions. The oxidation of the metal centers suggested the incorporation of oxygen into the crystal lattice of the initial compound, which was examined by X-ray photoelectron spectroscopy (XPS), X-ray diffraction. and Mössbauer spectroscopy.

**Figure 10:**
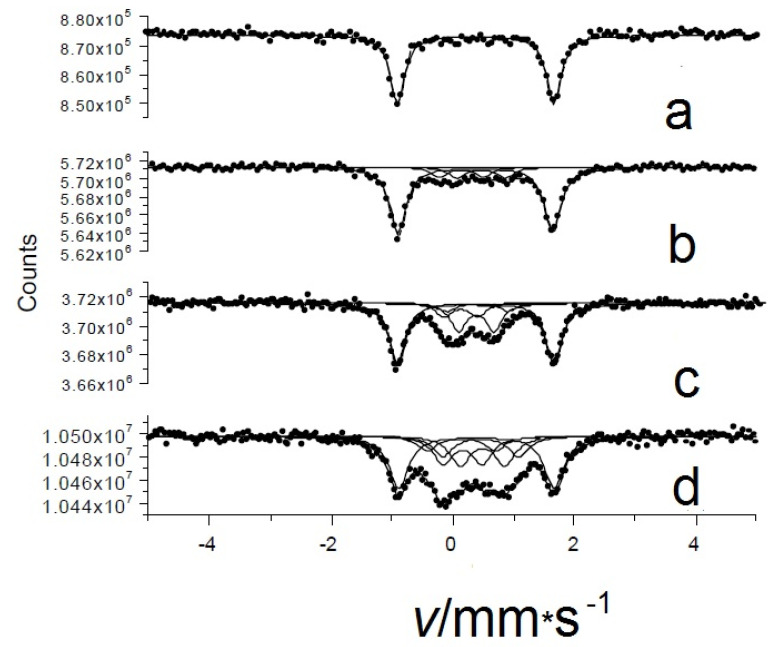
Room temperature Mössbauer spectra of pristine oxygen-free (**a**) and oxygen-loaded β-iron(II) phthalocyanines: oxygenated at room temperature in an O_2_/wet atmosphere for 14 days (**b**) at 50 °C in an O_2_/wet atmosphere for 14 days (**c**) and at 50 °C in an O_2_/dry atmosphere for 92 days (**d**).

As mentioned above, the initial compound in β-form has a metal center Fe^II^ with intermediate spin (S = 1, from [37]) in what is basically a square-planar environment with very long axial contacts with Pc molecules from adjacent layers. This creates a significant electric field gradient, EFG, leading to a large amount of quadrupole splitting Δ in the spectrum. In the first round of experiments, the room temperature Mössbauer spectra of the initial, pure, and oxygenated β-iron(II) phthalocyanines (displayed in Figure 10a) clearly show that the progressive oxidation of the starting compound over time led to the appearance of new chemical environments (Figure 10b–d). The new species are associated with different oxygenation products with much smaller quadrupole splitting values as compared to the quadrupole splitting, Δ = 2.54 ± 0.02 mm/s, of the initial pure β-iron(II) phthalocyanine (Figure 10a). The number and occurrence of the components depend on the oxygen uptake and increase with the amount of oxygen incorporated. After treatment with O_2_, the new spectral components had a significantly smaller quadrupole splitting Δ (Figure 10). Based on these observations, we tentatively ruled out the chemical oxidation process and suggest that oxygen can be incorporated between layers of the solid β-iron(II) phthalocyanine instead. Interestingly, this process does not occur in solution, which clearly indicates the role of a layered solid matrix. Regarding the location of oxygen, four different microenvironments were detected in the oxygenated β-form of iron (II) phthalocyanine [39] in our experiments. Among the components formed by oxygen uptake, the μ-oxo- and μ-peroxo-bridged species were identified.

**Figure 11:**
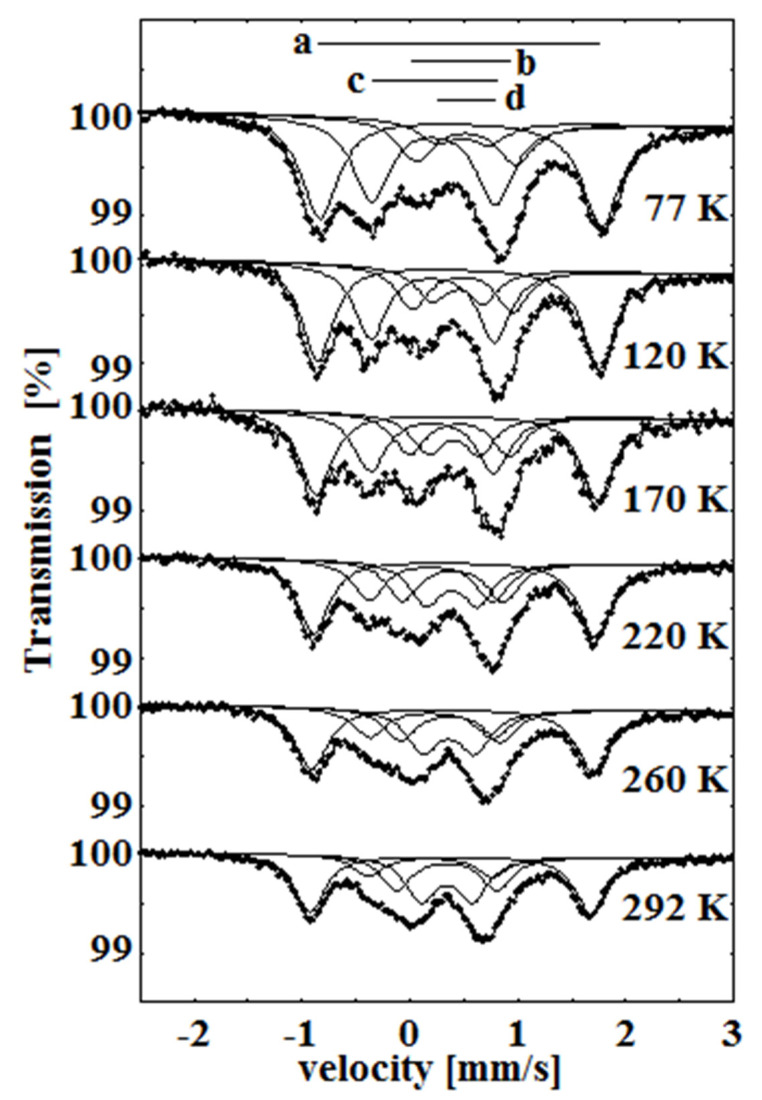
Temperature dependence of the Mössbauer spectra of β-iron phthalocyanines treated with oxygen in wet atmosphere at 50 °C for over 6 weeks. As the temperature increases, a transition is observed between the low-spin component *c* and the high-spin component *d*.

In our next study of the oxygenated β-iron(II) phthalocyanine, we observed the formation of an oxidized Fe^III^ species, and we also found the temperature-dependent spin transition (SCO) for one of the Fe^III^ components [40,41]. This process is illustrated in a series of overlaid spectra shown in Figure 11. When the temperature was raised from 77 to 292 K, the relative area of the low-spin component *c* decreased, and the area of the high-spin component *d* increased.

**Figure 12:**
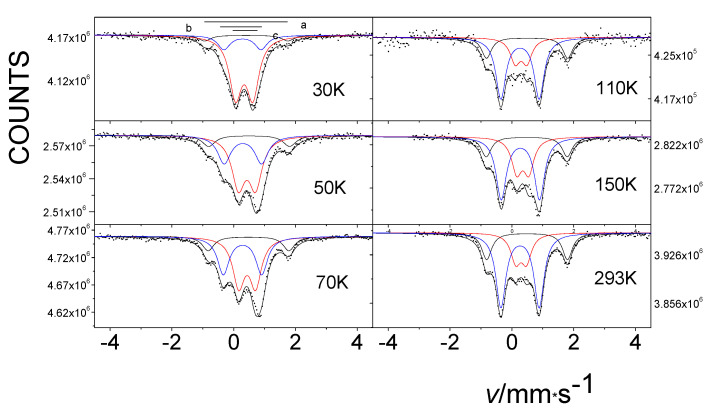
Temperature dependence of the Mössbauer spectra of β-iron phthalocyanines loaded with oxygen in an O_2_/H_2_O stream at 50 °C for 75 days. As the temperature increases, a transition is observed between the low-spin component *b* and the high-spin component *c*.

Continuing our research, we were able to see only the temperature-dependent spin transition occurring among oxygenated/oxidized species incorporated between the layers of the initial compound [42]. This is seen in the Mössbauer spectra displayed in Figure 12, showing the gradual increase in component *b* at the expense of component *c* with increasing temperature. The Mössbauer measurements indicated that the treatment of β-iron phthalocyanine in oxygen resulted in the formation of new species, corresponding to Fe^III^Pc oxygen adducts that are in a dynamic-temperature-dependent spin equilibrium (SCO). Thus, variable temperature Mössbauer spectra showed that as the temperature increased, the low-spin species transformed into the high-spin species (Figure 14) [42].

**Figure 13:**
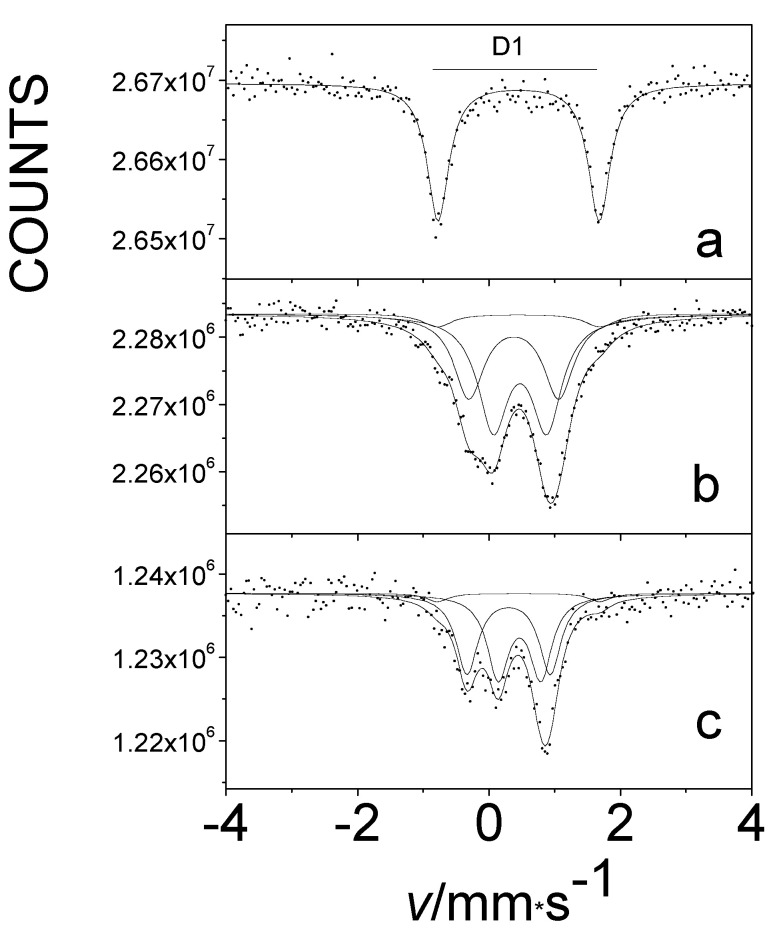
The Mössbauer spectra at 80 K of pristine oxygen-free (**a**) and oxygen-loaded α-iron phthalocyanines, treated at 50 °C in O_2_ for 75 days (**b**) and at 50 °C in O_2_/H_2_O for 75 days (**c**).

In light of our experience with β-iron phthalocyanines, we attempted to introduce oxygen into the α-iron-phthalocyanine polymorph under similar conditions. There is no chemical difference in bonding in either form, but there is a difference in crystal packing, which is reflected in a different angle of orientation of slipped π-stacks. In our earlier studies, it was impossible to insert any significant amount of oxygen between the layers of this solid compound. However, in our later work, we were able to achieve this. A comparison of the Mössbauer spectra of pure oxygen-free and oxygen-treated α-iron phthalocyanines at 80 K is shown in Figure 13. The D1 doublet, characteristic of the oxygen-free original sample (Figure 13a), represents the spectroscopic signature of iron(II) centers, and treatment with oxygen led to their dramatic decrease (Figure 13b,c). The corresponding Fe^II^ component was not detectable at room temperature after treatment. For all species in this series, the value of the isomer shift *δ* is remarkably similar, indicating that the oxidation state of metal did not change. We have for the first time succeeded in effectively oxygenating α-iron(II) phthalocyanine by an appropriate treatment with oxygen, such that all iron atoms have oxygen neighbors (Figure 13c) [43].

**Figure 14:**
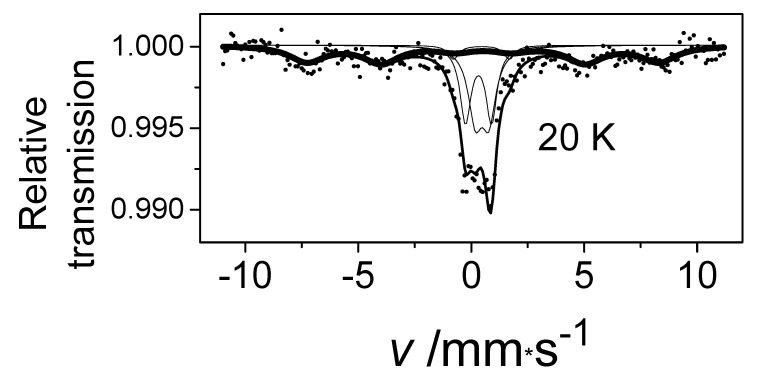
The Mössbauer spectrum of α-iron phthalocyanine loaded with oxygen in O_2_/H_2_O stream at 50 °C for 75 days measured at 20 K.

**Figure 15:**
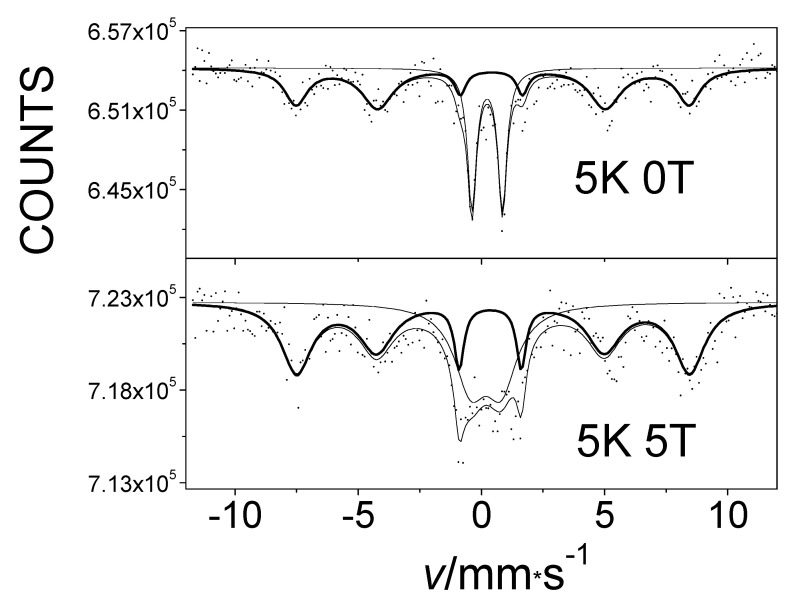
The Mössbauer spectra at 5 K of α-iron-phthalocyanine treated with oxygen in an O_2_/H_2_O stream at 50 °C for 75 days, without an external magnetic field (top) and at 5 T in an external magnetic field applied in parallel with gamma radiation direction (bottom).

Furthermore, we found that one of the components in the Mössbauer spectrum of the oxygen-treated α-iron(II)-phthalocyanine showed a typical six-line magnetic splitting pattern, as shown in Figure 14 [42,43]. To investigate the origin of the spectroscopic sextet that arose simply by cooling of this sample, the effect of an applied external magnetic field on the sample’s internal magnetic field was measured. These observations are well demonstrated by the Mössbauer effect measurements conducted at 5 K (Figure 15).

An analysis of spectra showed that the magnitude of the internal magnetic field remained unchanged when an external magnetic field was applied. This confirms the presence of an antiferromagnetic interaction in the studied system [43]. The antiferromagnetism of this sample was also verified by measurements of its magnetic susceptibility [43].

**Figure 16:**
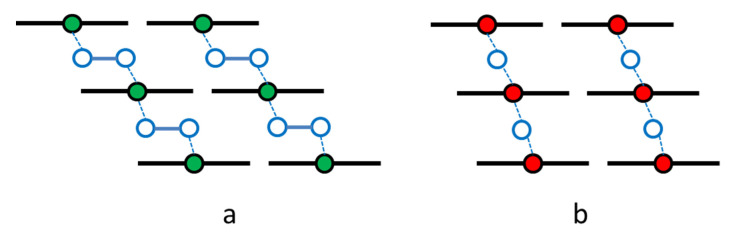
Models of the oxygen binding that bridges iron centers, m-peroxo Fe^II^Pc (**a**) and m-oxo Fe^III^Pc (**b**), in oxygen-loaded solid iron phthalocyanines.

In summary, the antiferromagnetic coupling of magnetism observed at low temperatures in the highly oxygen-loaded α-iron phthalocyanines was explained by a super-exchange interaction between the long μ-peroxo – Fe –O–O– Fe– zigzag chains connecting layers of complexes (Figure 16) [43]. We realize that this model opposes data on the existing and well-known μ-oxo Fe–O–Fe dimers and polymers observed in the chemistry of porphyrins [44], phthalocyanines, and texaphyrins [45]. Additional studies involving oxygen isotope labeling and Raman spectroscopy are necessary to elucidate the structures displayed in Figure 16 in more detail. 

## 4. Conclusions

This review presents the results of some of our studies of totally different iron-containing compounds and systems using ^57^Fe Mössbauer spectroscopy, which is shown to be a powerful tool for structural and magnetic investigations.

## Figures and Tables

**Table 1:** Hyperfine field values and N-Fe-N bond angles in quasi-linear Fe^(II)^ amido-compounds.

Compound	Fe(L2)_2_	Fe(L5)_2_	Fe(L4)_2_	Fe(L3)_2_
N-Fe-N angle, ^o^	180	179	169	141
*B*_int_, Tesla	122	105	92	67

## Data Availability

Data is contained within the article or cited references.
